# Sustainable Triazine-Based Dehydro-Condensation Agents for Amide Synthesis

**DOI:** 10.3390/molecules26010191

**Published:** 2021-01-02

**Authors:** Roberto Sole, Vanessa Gatto, Silvia Conca, Noemi Bardella, Andrea Morandini, Valentina Beghetto

**Affiliations:** 1 Dipartimento di Scienze Molecolari e Nanosistemi, Università Ca’ Foscari di Venezia, Via Torino 155, 30172 Venezia, Italy; roberto.sole@unive.it (R.S.); silvia.conca@unive.it (S.C.); noemi.bardella@unive.it (N.B.); andrea.morandini@unive.it (A.M.); 2 Crossing srl, Viale della Repubblica 193/b, 31100 Treviso, Italy; vanessa.gatto@unive.it

**Keywords:** CDMT, triazines, amide synthesis, sustainable dehydro-condensation agents

## Abstract

Conventional methods employed today for the synthesis of amides often lack of economic and environmental sustainability. Triazine-derived quaternary ammonium salts, e.g., 4-(4,6-dimethoxy-1,3,5-triazin-2-yl)-4-methylmorpholinium chloride (DMTMM(Cl)), emerged as promising dehydro-condensation agents for amide synthesis, although suffering of limited stability and high costs. In the present work, a simple protocol for the synthesis of amides mediated by 2-chloro-4,6-dimethoxy-1,3,5-triazine (CDMT) and a *tert*-amine has been described and data are compared to DMTMM(Cl) and other CDMT-derived quaternary ammonium salts (DMT-Ams(X), X: Cl^−^ or ClO_4_^−^). Different *tert*-amines (Ams) were tested for the synthesis of various DMT-Ams(Cl), but only DMTMM(Cl) could be isolated and employed for dehydro-condensation reactions, while all CDMT/*tert*-amine systems tested were efficient as dehydro-condensation agents. Interestingly, in best reaction conditions, CDMT and 1,4-dimethylpiperazine gave *N*-phenethyl benzamide in 93% yield in 15 min, with up to half the amount of *tert*-amine consumption. The efficiency of CDMT/*tert*-amine was further compared to more stable triazine quaternary ammonium salts having a perchlorate counter anion (DMT-Ams(ClO_4_)). Overall CDMT/*tert*-amine systems appear to be a viable and more economical alternative to most dehydro-condensation agents employed today.

## 1. Introduction

The synthesis of amides is among the most important reactions in organic chemistry and biochemistry because of the widespread occurrence of amides in pharmaceuticals, peptides, biologically active compounds, industrial polymers, detergents, and lubricants [1,2,3,4,5,6]. Consequently, many different physical [7] and chemical protocols have been developed for the synthesis of amides by dehydro-condensation of a carboxylic acid and an amine [8,9,10,11,12,13]. Catalytic routes for the preparation of amides have also been reported with the intent to increase atom efficiency of the process [2,14]. An alternative metal-free synthetic route for the formation of amides has been recently proposed by Zhang et al. [15] by coupling reaction of formamide with different carboxylic acids.

Albeit the strategic importance of this class of compounds, most methods commonly employed for the synthesis of amides still lack both in economic and environmental sustainability, have low atom efficiency, generate large quantities of hazardous waste products, and require complicated purification steps [16,17,18].

**Figure 1:**
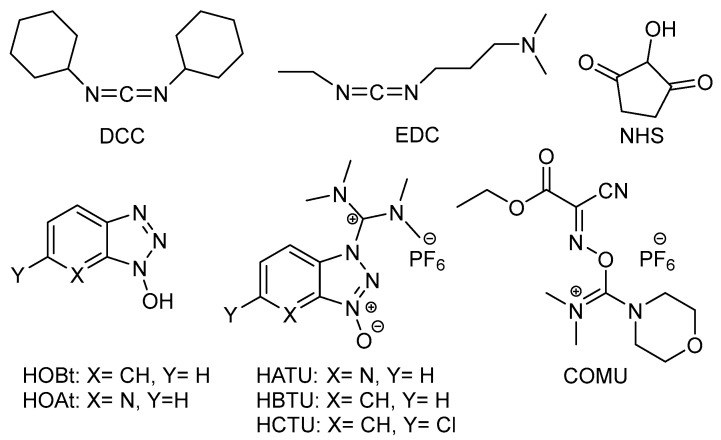
Chemical structures of various dehydro-condensation agents.

In this frame, dehydro-condensation reactions are a well-known and consolidated protocol for the synthesis of amides, although toxic dehydro-condensation agents are often used to promote the reaction [17,19]. For example, carbodiimides such as dicyclohexylcarbodiimide (DCC) or 1-ethyl-3-(3-dimethylaminopropyl)carbodiimide chlorohydrate (EDC) employed in combination with *N*-hydroxysuccinimide (NHS), have frequently been employed for the scope (Figure 1) [20,21,22,23].

Hydroxybenzotriazole (HOBt) [24,25,26], 1-hydroxy-7-azabenzotriazole (HOAt) [27,28,29], and uronium or guanidinium salt derivatives such as, e.g., hexafluorophosphate azabenzotriazole tetramethyl uronium (HATU), hexafluorophosphate benzotriazole tetramethyl uronium (HBTU), hexafluorophosphate chlorobenzotriazole tetramethyl uronium (HCTU), and 1-cyano-2-ethoxy-2-oxoethylidenaminooxy)-dimethylamino-morpholino-carbenium hexafluorophosphate (COMU) (Figure 1) have also been efficiently used to promote carbodiimide-mediated peptide coupling reactions [18,30], although with scarce environmental and economic sustainability [9,10,31]. In fact, DCC and EDC lead to the formation of toxic by-products [17], while uronium derivatives may contain highly harmful hydrazide impurities [19].

More stringent rules regulating the use and disposal of organic solvents and increasingly restrictive EU directives on hazardous substances make the quest for greener and more efficient products and processes increasingly important [17,18].

**Figure 2:**
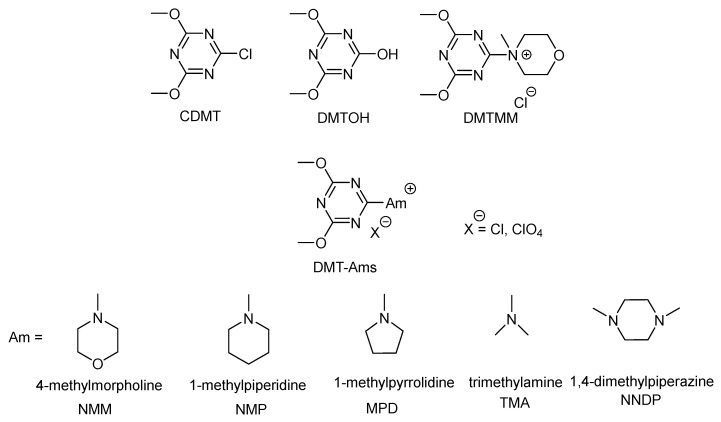
Chemical structures of 2-chloro-4,6-dimethoxy-1,3,5-triazine (CDMT), 2,4-dimethoxy-6-hydroxy-1,3,5-triazine (DMTOH), 4-(4,6-dimethoxy-1,3,5-triazin-2-yl)-4-methylmorpholinium chloride (DMTMM) and 2-(4,6-dimethoxy-1,3,5-triazinyl)trialkyl ammonium salts (DMT-Ams).

In this prospective, quaternary ammonium salts of 2-halo-4,6-dialkoxy-1,3,5-triazines, e.g., 4-(4,6-dimethoxy-1,3,5-triazin-2-yl)-4-methylmorpholinium chloride (DMTMM(Cl)), (see Figure 2), represent a valid alternative for the formation of amide bonds both in organic and water solvent [20,21,32,33,34,35,36,37,38,39]. In the last years, DMTMM(Cl), commonly known as DMTMM, has found many applications as dehydro-condensation agent for the synthesis of amides and esters [36,40], collagen cross-linking [7,21,38], preparation of carboxymethylcellulose based films [39], amine grafting on hyaluronan [20,40,41], and peptide synthesis [42]. Additionally, the synthesis of a library of 2-(4,6-dimethoxy-1,3,5-triazinyl)trialkyl ammonium salts (DMT-Ams(X), X: Cl^−^ or ClO_4_^−^) and their activity as dehydro-condensation agents for the synthesis of amides, has been reported by Kunishima et al. [43] (Figure 2). This work is a milestone in the understanding of triazine based-condensation agents and their application in various fields of chemistry. Further interesting feature is that DMTMM and DMT-Ams(X) all degrade forming 2,4-dimethoxy-6-hydroxy-1,3,5-triazine (DMTOH) and the corresponding tertiary amine hydrochloric salt, which are nontoxic. Moreover, DMTOH can be recovered and recycled to produce 2-chloro-4,6-dimethoxy-1,3,5-triazine (CDMT) or DMTMM (Figure 2) [37]. To date, one of the main limitations to large-scale use of DMTMM and DMT-Ams(X) remains their reduced stability in solution [43,44].

All this in mind, we believe that the use of isolated triazine quaternary ammonium salts could be overcome by direct addition of a 2-halo-4,6-dialkoxy-1,3,5-triazine and a *tert*-amine in the reaction mixture. This straightforward methodology would avoid the synthesis of isolated DMT-Ams(X), reduce production complexity, and solvent use and allow to achieve a library of dehydro-condensation agents, modulated according to specific requirements. Nowadays, a few data are available on dehydro-condensation reactions carried out with similar protocols [43,45] and often referring to very specific substrates in rather limited reaction conditions [46,47,48,49,50]. Only recently, Kitamura [34] and Kunishima [35] reported a similar protocol for amide condensation reactions, albeit in the presence of amido- or imido-chlorotriazines. To the best of our knowledge, no in-depth study has been reported on the advantages derived from the use of different CDMT/*tert*-amine systems compared to isolated DMT-Ams(X).

Our research group has long been involved in the study of innovative sustainable processes for fine chemistry [51,52,53,54]. Recently, our studies have focused on the development of new dehydro-condensation agents. Therefore, in this work we wish to report a systematic comparison between the efficiency of CDMT/*tert*-amine systems and isolated DMT-Ams(X) for the synthesis of amides.

Moreover, the possible advantages derived from the use of CDMT/*tert*-amine systems were also analyzed.

## 2. Results and Discussion

The activity of CDMT/*tert*-amine systems for dehydro-condensation reactions has been compared with the activity of the corresponding isolated DMT-Ams(X). DMT-Ams(X) have been synthesized according to the standard protocol reported in the literature [43,55,56,57]. For example, DMTMM was prepared by reaction of one equivalent of 2-chloro-4,6-dimethoxy-1,3,5-triazine (CDMT) and 1.2 equivalents of *N*-methyl morpholine (NMM) in THF at room temperature for 1 h. After work up, DMTMM was recovered in 85–88% yield.

**Scheme 1:**
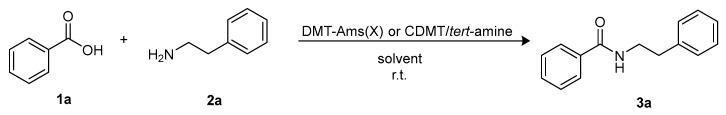
Dehydro-condensation reaction for the synthesis of amide **3a**.

In the present work, the CDMT/*tert*-amine protocol foresees dissolution of 1.3 mmoles of an acid (e.g., **1a**) in the reaction solvent, followed by addition (within a few seconds) of 1.3 mmoles of CDMT, 1.3 mmoles of a *tert*-amine, and 1.2 mmoles of an amine (e.g., **2a**) (Scheme 1).

The yield in amide **3a** was monitored by GLC after 15 min and 1 h (see e.g., entry 1 of Table 1). For a set of preliminary experiments, the reaction mixture was monitored by GLC also after 24 h; since no significant difference was observed between the yield in amide **3a** after 1 and 24 h, unless otherwise stated, all further reactions were monitored within 1 h.

### 2.1. Influence of the Solvent on Dehydro-Condensation Reactions

Preliminarily investigations on the CDMT/*tert*-amine system were carried out by using NMM as amine. The influence of the solvent on the CDMT/NMM dehydro-condensation activity was tested employing benzoic acid **1a** and phenylethylamine **2a** as standard substrates (Scheme 1). Relevant data are reported in Table 1 and compared with data from analogous reactions carried out in the presence of isolated DMTMM. All data reported in Table 1 are the mean values achieved for a set of at least three experiments monitored after 15 min and 1 h.

Interestingly, after 15 min, the CDMT/NMM system afforded, in most of the organic solvents used, the amide **3a** in equivalent or even better yields compared to DMTMM. In CH_3_OH, almost quantitative yield of **3a** was measured both with DMTMM (98%) and CDMT/NMM (93%) by 15 min (entries 3 and 4, Table 1). Noteworthy, the use of an alcohol (entries 3–6, Table 1) as reaction solvent achieved **3a** (≥90%) with very high selectivity, both by CDMT/NMM system and isolated DMTMM, and only traces of the corresponding ester were detected. These data are in agreement with Kunishima work [58], where the rate of nucleophilic attack of 2-phenylethylamine to 3-phenylpropionic acid (aminolysis) was found to be approximately 2 × 10^4^ times faster than methanolysis. This is a significant advantage compared to other dehydro-condensation agents such as EDC/NHS for which yields and selectivity in similar reaction conditions are known to be poor [6,20]. Moreover, the difference in reactivity of the amine functionality, compared to, e.g., -OH or -SH groups, allows to carry out chemoselective dehydro-condensation reactions for the synthesis of amides, starting from carboxylic acids or amines bearing also -OH or -SH functionalities, as widely reported in the literature [58,59,60,61]. Additionally, when the amine contains both a hydroxyl and an amine functional group, no ester by-product is observed confirming that the reactivity of the amine functional group is by far much higher than that of the -OH group so that no ester is formed in the presence of DMTMM [46,58,61] and other 1,3,5-triazine-based dehydro-condensation agents [57]. In comparison to catalytic amidation reactions, triazine-derived dehydro-condensation agents proceed well also in relatively nonpolar solvents, which still today limits wide application of catalytic amidation reactions [4].

In fact, in toluene as solvent, CDMT/NMM led to very good yield of **3a** (90%) after 1 h, higher than in the presence of DMTMM (entries 13 and 14, Table 1). On the other hand, significantly lower yields in amide **3a** where obtained in water as reaction solvent (entries 15 and 16 of Table 1) probably due to low water solubility of the substrates.

Thus, according to data reported in Table 1, activity and selectivity of CDMT/NMM system and isolated DMTMM were shown to be very similar. The use of alcohols promoted best performances for both dehydro-condensation systems tested, hence, further experiments were carried out in CH_3_OH.

### 2.2. Influence of the Tert-Amine on Dehydro-Condensation Reactions

**Figure 3:**
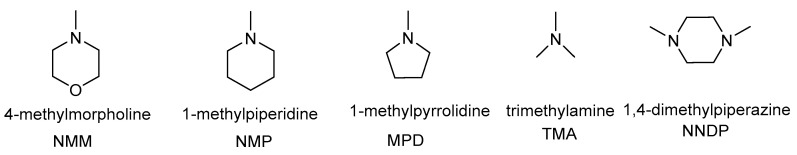
*Tert*-amines used in combination with CDMT.

Spurred by the positive results obtained, further dehydro-condensation reactions for the synthesis of **3a** were carried out in the presence of CDMT and different tertiary *N*-methyl-substituted aliphatic amines (see Figure 3) employing methanol as solvent. Since, the synthesis of quaternary ammonium salts prepared from CDMT and *N*-ethyl tertiary aliphatic or aromatic amines has been extensively studied in the literature [43], showing in most cases very unsatisfactory results, *N*-ethyl *tert*-amines were not investigated in this work.

**Scheme 2:**
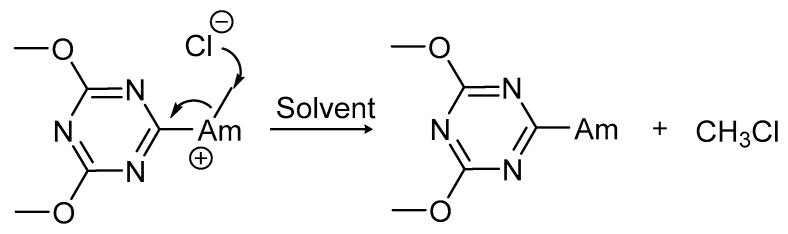
The 2-(4,6-dimethoxy-1,3,5-triazinyl)trialkyl ammonium salts (DMT-Ams) demethylation.

Data collected for the synthesis of **3a** in the presence of different CDMT/*tert*-amine systems were compared, when possible, with corresponding dehydro-condensation data achieved in the presence of isolated DMT-Ams(X) (see Table 2). In fact, comparison of the activity of DMT-Ams(X) and CDMT/*tert*-amine systems as dehydro-condensation agents was not always possible since, depending on the *tert*-amine used, the isolation of the DMT-Ams(X) may be problematic, due to instability of the corresponding quaternary ammonium salt and formation of a triazine derivative, which is totally inactive as dehydro-condensation agent (see Scheme 2) [57]. As a matter of fact, only DMTMM was isolated in good yield while all other DMT-Ams(Cl) were highly unstable and could not be isolated.

All CDMT/*tert*-amine systems tested were active as dehydro-condensation agents, as reported in Table 2. Similarly to CDMT/NMM, the CDMT/TMA system allowed to obtain **3a** with 96% yield in 1 h (entry 5, Table 2).

Moderately lower yields of amide **3a** were obtained with CDMT/NMP (49%, entry 9, Table 2) or CDMT/MPD (65%, entry 13, Table 2). CDMT was also tested in combination with 1,4-dimethylpiperazine (NNDP, entries 17–19, Table 2) to verify if double quaternarization of the two tertiary amine functionalities present on NNDP could allow to reduce the amount of *tert*-amine required. In fact, when a 1/1 molar ratio of CDMT/NNDP was employed, yields in **3a** achieved were comparable to the ones obtained with DMTMM and CDMT/NMM (compare entries 1, 2, and 17, Table 2). Notably, when the CDMT/NNDP molar ratio was decreased to 1/0.75 (entry 18, Table 2) and 1/0.5 (entry 19, Table 2), excellent yields of **3a** were still achieved, reducing up to half the amount of *tert*-amine employed. Adversely, DMT-NNDP(X) could not be isolated either with Cl^−^ or ClO_4_^−^ counter anion (see below). From this first screening, higher versatility of CDMT/*tert*-amine system compared to isolated DMT-Ams(X) clearly appeared.

In order to compare the activity of CDMT/*tert*-amine systems with isolated DMT-Ams(X), stability problems, which strongly limited the library of isolated DMT-Ams(Cl) available, were partially overcome by counter anion exchange of the Cl^−^ atom with ClO_4_^−^, BF_4_^−^, and PF_6_^−^ [43,55]. Nevertheless, since BF_4_^−^ and PF_6_^−^ gave scantly soluble quaternary ammonium salts, only DMT-Ams(ClO_4_) were further synthesized and their activity for dehydro-condensation reactions was compared with CDMT/*tert*-amine systems. Data reported in Table 2 revealed that most of the isolated DMT-Ams(ClO_4_) tested for the dehydro-condensation reaction of **1a** and **2a** gave good yields in **3a**, comparable to those achieved with the corresponding CDMT/*tert*-amine systems. Nevertheless, it should be considered that the synthesis of isolated DMT-Ams(ClO_4_) required the use of organic solvents (THF) and a stochiometric amount of a perchlorate salt, reducing their overall sustainability.

Moreover, counter anion exchange in the presence of DMTMM strongly reduced the activity of the corresponding isolated perchlorate salt, and the yield in **3a** decreased from 99% with DMTMM (entry 2, Table 2) to 47% with DMTMM(ClO_4_), by 1 h (entry 4, Table 2). This behavior may be attributed to two different but correlated phenomena: substitution of the counter anion generally increased the stability of the isolated ammonium salt but at the same time, it decreased the solubility of the quaternary ammonium salt and consequently its reactivity as dehydro-condensation agent [55]. Thus, by counter anion exchange, DMTMM(ClO_4_) could be isolated but, in CH_3_OH as solvent, had reduced solubility and reactivity as dehydro-condensation agent.

In principle, counter anion exchange should not influence the activity of CDMT/*tert*-amine systems, used according to the procedure reported in this work, since no isolation of DMT-Ams(X) was required and thus stability problems should not be encountered. In fact, in agreement with this hypothesis and according to data reported in Table 2, the addition of NaClO_4_ in most cases did not significantly alter the activity of the CDMT/*tert*-amine system. Comparing CDMT/TMA (entries 5 and 7, Table 2), CDMT/NMP (entries 9 and 11, Table 2), and CDMT/MPD (entries 13 and 15, Table 2) systems with Cl^−^ or ClO_4_^−^ anion, yields in **3a** were approximately equivalent.

However, for CDMT/NMM/NaClO_4_ system, the activity in dehydro-condensation reaction of **1a** and **2a** significantly decreased and yield of **3a** were lower than the ones achieved with CDMT/NMM, even after 1 h (entries 1 and 3, Table 2).

**Figure 4:**
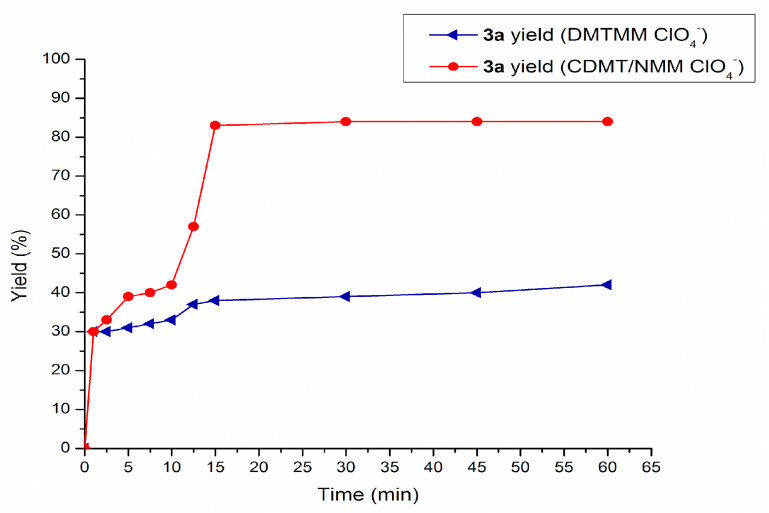
Kinetic profile of dehydro-condensation reaction between **1a** and **2a** in the presence of CDMT/*N*-methyl morpholine (NMM)/ClO_4_ and DMTMM(ClO_4_).

Since data reported in Table 1 and Table 2 showed in most cases very small differences in yields of **3a** after 15 min and 1 h, in order to better compare the reactivity of the CDMT/*tert*-amine system to the DMT-Ams(X) one, a kinetic study was carried out (Figure 4). Data reported in Figure 4 show the kinetic profile for the yield in **3a**, monitored by GLC over a period of 1 h at room temperature, in the presence of CDMT/NMM/ClO_4_ system or isolated DMTMM(ClO_4_) (entries 3–4, Table 2).

Notably, formation of **3a** was already evidenced after 1 min from the start of the reaction with both dehydro-condensation systems, and after 5 min significant differences in the rate of formation of **3a** were evident between CDMT/NMM/ClO_4_ system and isolated DMTMM(ClO_4_). The second considerable gap occurred after 10 min, as the CDMT/NMM/ClO_4_ system proceeded with an exponential type trend, while DMTMM(ClO_4_) maintained an approximately linear trend. Finally, in both cases, a plateau was reached after 15 min.

**Scheme 3:**
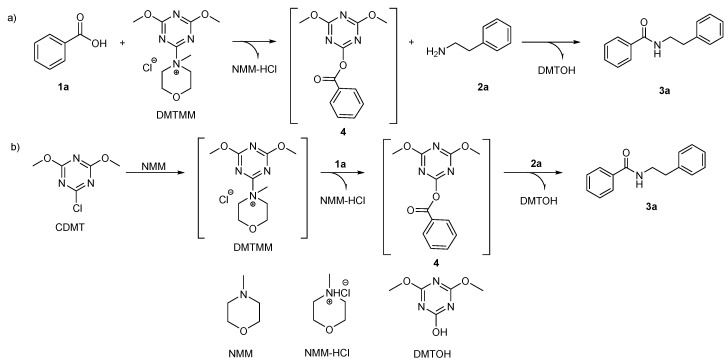
Possible known pathways for amidation of **1a** and **2a** in the presence of 4-(4,6-dimethoxy-1,3,5-triazin-2-yl)-4-methylmorpholinium chloride (DMTMM) (**a**) or 2-chloro-4,6-dimethoxy-1,3,5-triazine (CDMT) (**b**) /*N*-methyl morpholine (NMM) system.

Data gathered in Table 2 and Figure 4 contributed to give deeper insight into the activity and versatility of CDMT/*tert*-amine system compared to isolated DMT-Ams(X) for carboxylic acid-amine dehydro-condensation reactions, according to the protocol reported in this work. Counter anion experiments evidenced differences between isolated DMT-Ams(X) and CDMT/*tert*-amine systems, possibly ascribable to differences in the reaction pathway. According to the literature [56,57,62] when isolated DMTMM is employed as condensation agent, the reaction mechanism initially foresees the reaction between the carboxylic acid and DMTMM, forming an activated ester (**4**), which then undergoes attack by an amine to give the corresponding amide as illustrated in Scheme 3 pathway **a**. Alternatively, literature data report a two steps protocol, foreseeing reaction of CDMT/*tert*-amine in the reaction solvent in the presence of a carboxylic acid for approximately 1 h, followed by addition of a primary amine (Scheme 3 pathway **b**) [43]. According to literature data, it is generally assumed that, in these reaction conditions, once CDMT and the *tert*-amine are added to the reaction solvent, the corresponding quaternary ammonium salt is formed prior to dehydro-condensation reaction.

Preliminary experimental evidences collected by ^1^H NMR spectroscopy (see Supporting Information) seem to indicate that the protocol reported in this work for the use of CDMT/*tert*-amine systems differs from previously reported mechanisms and may not lead to the formation of DMT-Ams(X) as reported in Scheme 3, but possibly to the straightforward formation of the intermediate active ester (**4**), which according to the literature is supposed to be the rate-determining step, thus boosting the efficacy of the derived dehydro-condensation agents [58,63]. Further experiments are ongoing to gain deeper insight into this reaction mechanism.

### 2.3. CDMT/Tert-Amine Activity for the Dehydro-Condensation of Various Primary Amines and Carboxylic Acids

**Figure 5:**
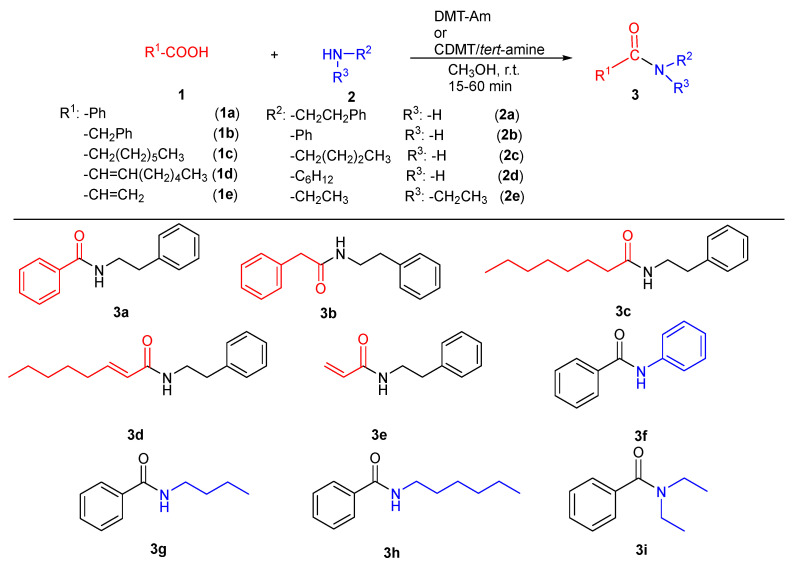
Reaction between different acids (**1a–e**) and different amines (**2a–e**) in the presence of various CDMT/*tert*-amine systems.

To widen the scope of the CDMT/*tert*-amine system, dehydro-condensation reactions for the synthesis of different amides were tested (see Figure 5) ranging from aliphatic, aromatic, sterically hindered, and α,β-unsaturated acids.

Only *tert*-amines that gave best results, as reported in Table 2, were further investigated (NMM, NNDP, and TMA). Primary and secondary aliphatic amines as well as aniline could be condensed to give the corresponding amides (see Table 3). Best yields in **3b** were obtained with CDMT/NMM (87% in 1 h, entry 4, Table 3), although with slightly lower yield than for **3a** (95%, entry 1, Table 3) probably due to reduced nucleophilicity of the carboxyl group of **1b** compared to **1a**, which disfavors the formation of the active ester.

Similar charge effects were observed for the synthesis of **3c** and **3d** (see entries 7 and 10, Table 3). **1e** was tested only with CDMT/NMM system giving very low yields (38% in 1 h, entry 13, Table 3).

Further dehydro-condensation reactions of **1a** and amines **2b**–**2e** are reported in Table 4. When amine **2b** was used, a specular behavior to the one reported for **3b** was observed, since the presence of a phenyl ring vicinal to the amine functionality increases electron density on the nitrogen atom reducing the overall dehydro-condensation yield in **3f**.

Aliphatic amine **2c** gave very good dehydro-condensation yields in **3g**, above 75%, both with CDMT/NMM and CDMT/TMA (entries 4 and 6, Table 4).

Sterical hindrance was supposed to be the cause of moderately lower yields in **3h**. Secondary aliphatic amines (**2e**) are known to be less reactive and, in fact, yields in the corresponding amide (**3i**) were lower (entries 10–12, Table 4).

## 3. Materials and Methods

All chemicals were purchased from Sigma Aldrich Co. (St. Louis, MO). GC analyses were carried out on an Agilent 6850A Gas chromatograph (HP1 capillary column). GC–MS analyses were performed on an Agilent MS Network 5937 (HP5 capillary column). Mesitylene was used as internal standard. ^1^H and ^13^C {^1^H} NMR spectra were recorded on a Bruker AVANCE 300 spectrometer operating at 300.21 and 75.44 MHz. The chemical shift values of the spectra are reported in δ units with reference to the residual solvent signal.

Unless otherwise reported, DMT-Ams(ClO_4_) were synthesized from CDMT with the corresponding tertiary amine and NaOCl_4_ according to the synthetic procedure reported in the literature [43,53]. All products, **3a-3i**, were characterized and purity was confirmed by comparison to the literature data [64,65,66,67,68,69,70].

### 3.1. Synthesis of DMTTMA(ClO_4_)

TMA (75 mg, 1.27 mmol) was added to a solution of CDMT (200 mg, 1.14 mmol) and NaClO_4_ (155 mg, 1.27 mmol) in THF (4 mL) at 25 °C. After stirring for 1 h, the precipitate was collected, washed with THF, and dried under reduced pressure to give DMTTMA(ClO_4_)^−^ as a white solid (340 mg, yield 99%). m.p. 163–168 °C; ^1^H NMR (300 MHz, D_2_O, 25 °C): δ = 4.21 (s, 6H), 3.65 ppm (s, 9H); ^13^C NMR (75 MHz, D_2_O, 25 °C): δ = 175.2, 173.6, 58.6, 55.7 ppm; IR (KBr): ṽ = 1606, 1531, 1485, 1375, 1052, 825, 624 cm^−1^.

### 3.2. General Procedure for Dehydro-Condensation Reactions with CDMT/Tert-Amine System

Acid **1a** (158.7 mg, 1.3 mmol) was dissolved in a solvent (MeOH, 6 mL) and subsequently CDMT (228.2 mg, 1.3 mmol), NMM (131.5 mg, 1.3 mmol), and amine **2a** (145.4 mg, 1.2 mmol) were added. After 15 min and 1 h, yield in amide **3a** was monitored by GLC using mesitylene as internal standard. To isolate **3a**, the reaction mixture was filtered and the yellowish solid recovered was dissolved in CH_2_Cl_2_ and extracted with water (3 × 30 mL). The combined organic phases were dried over MgSO_4_ filtered and concentrated in vacuo to give **3a** as a white solid.

### 3.3. General Procedure for Dehydro-Condensation Reaction with Isolated DMTMM

Acid **1a** (158.7 mg, 1.3 mmol) was dissolved in solvent (MeOH, 6 mL) and subsequently DMTMM (359.7 mg, 1.3 mmol) and amine **2a** (145.4 mg, 1.2 mmol) was added. After 15 min and 1 h, yield in amide **3a** was monitored by GLC using mesitylene as internal standard. The procedure to isolate **3a** was the same as abovementioned.

#### NMR Characterization of Products **3a**–**3i**

***N*-phenylethyl-benzamide** (**3a**): ^1^H NMR (300 MHz, CDCl_3_, 25 °C): δ = 7.79–7.60 (m, 2H), 7.57–7.08 (m, 8H), 6.10 (bs, 1H), 3.73 (q, *J* = 6.6 Hz, 2H), 2.95 ppm (t, *J* = 6.9 Hz, 2H); ^13^C NMR (75 MHz, CDCl_3_, 25 °C): δ = 167.4, 138.4, 134.5, 132.0, 128.8, 128.6, 127.7, 126.5, 125.9, 41.1, 34.9 ppm.

***N*-phenethyl-2-phenylacetamide** (**3b**): ^1^H NMR (300 MHz, CDCl_3_, 25 °C): δ = 7.35–7.28 (m, 2H), 7.28–7.21 (m, 1H), 7.21–7.17 (m, 1H), 7.06–7.02 (m, 1H),5.52 (bs, 1H), 3.54 (s, 2H), 3.47 (c, *J* = 6.7 Hz, 2H), 2.75 ppm (t, *J* = 6.7 Hz, 2H); ^13^C NMR (75 MHz, CDCl_3_, 25 °C): δ = 171.2, 138.9, 135.1, 129.6, 129.2, 128.2,128.2, 127.1, 126.4, 43.8, 40.9, 35.6 ppm.

***N*-Phenethyloctanamide** (**3c**) ^1^H NMR (300 MHz, CDCl_3_, 25 °C): δ = 7.30 (t, *J* = 7.3 Hz, 2H), 7.25–7.21 (m, 1H), 7.21–7.14 (m, 2H), 5.57 (br. s, 1H), 3.51 (q, *J* = 6.8 Hz, 2H), 2.81 (t, *J* = 6.8 Hz, 2H), 2.11 (t, *J* = 7.6 Hz, 2H), 1.62–1.53 (m, 2H), 1.31–1.20 (m, 8H), 0.87 ppm (t, *J* = 6.7 Hz, 3H); ^13^C NMR (75 MHz, CDCl_3_, 25 °C): δ = 173.3, 139.1, 128.9, 128.7, 126.6, 40.6, 36.9, 35.8, 31.8, 29.3, 29.1, 25.9, 22.7, 14.2 ppm.

***N*-(2-phenylethyl)oct-2-enamide** (**3d**) ^1^H NMR (300 MHz, CDCl_3_, 25 °C): δ = 7.30 (t, *J* = 7.3 Hz, 2H), 7.25–7.21 (m, 1H), 7.21–7.14 (m, 2H), 5.57 (br. s, 1H), 3.51 (q, *J* = 6.8 Hz, 2H), 2.81 (t, *J* = 6.8 Hz, 2H), 2.11 (t, *J* = 7.6 Hz, 2H), 1.62–1.53 (m, 2H), 1.31–1.20 (m, 8H), 0.87 ppm (t, *J* = 6.7 Hz, 3H); ^13^C NMR (75 MHz, CDCl_3_, 25 °C): δ = 173.3, 139.1, 128.9, 128.7, 126.6, 40.6, 36.9, 35.8, 31.8, 29.3, 29.1, 25.9, 22.7, 14.2 ppm.

**2-phenylethyl acrylamide** (**3e**) ^1^H NMR (300 MHz, CDCl_3_, 25 °C): δ = 7.27–7.20 (m, 5H), 6.92–6.87 (m, 1H), 6.18–6.12 (m, 1H), 3.44(t, *J* = 5.1 Hz, 2H), 2.72 (t, *J* = 5 Hz, 2H), 2.22–2.18 (q, *J* = 6.8 Hz, 2H), 1.38–1.25 (m, 6H). 0.90 ppm (m, 3H); ^13^C NMR (75 MHz, CDCl_3_, 25 °C): δ = 166.8, 142.1, 138.8, 128.7, 128.6, 125.4,122.1, 40.6, 35.6, 33.8, 32.9, 31.8, 28.5, 22.7, 14.3 ppm.

**Phenylbenzamide** (**3f**) ^1^H NMR (300 MHz, CDCl_3_, 25 °C): δ = 7.13–7.87 ppm (m, 10H); ^13^C NMR (75 MHz, CDCl_3_, 25 °C): δ = 165.8. 138.0 135.0 131.9, 129.1, 128.8, 127.1, 124.6, 120.3 ppm.

***N*-Butylbenzamide** (**3g**) ^1^H NMR (300 MHz, CDCl_3_, 25 °C): δ = 7.74–7.78 (m, 2H), 7.47–7.51 (m, 1H), 7.40–7.45 (m, 2H), 6.14 (br s, 1H), 3.46 (dt, 2H, *J* = 6.8, 6.4 Hz), 1.61 (tt, 2H, *J* = 7.2, 7.2 Hz), 1.42 (qt, 2H, *J* = 7.2, 7.6 Hz), 0.96 ppm (t, 3H, *J* = 7.4 Hz); ^13^C NMR (75 MHz, CDCl_3_, 25 °C): δ = 167.6, 134.9, 131.3, 128.5, 126.8, 39.8, 31.8, 20.2, 13.8 ppm.

***N*-Hexylbenzamide** (**3h**) ^1^H NMR (300 MHz, CDCl_3_, 25 °C): δ = 7.77–7.75 (m, 2H), 7.39–7.47 (m, 3H), 6.42 (br s, 1H, NH), 3.43 (d, *J* = 5.5 Hz, 2H), 1.60 (d, *J* = 6.5 Hz, 2H), 1.36 (s, 2H), 1.31(d, *J* = 2.5 Hz, 4H), 0.88 ppm (d, *J* = 6.0 Hz, 3H); ^13^C NMR (75 MHz, CDCl_3_, 25 °C): δ = 167.5, 134.8, 131.2, 128.4, 126.8, 40.1, 31.5, 29.6, 26.6, 22.5, 14.0 ppm.

***N*,*N*-Diethylbenzamide** (**3i**). ^1^H NMR (300 MHz, CDCl_3_, 25 °C): δ = 7.46−7.30 (m, 5H), 3.40 (br s, 4H), 1.17 ppm (br s, 6H); ^13^C NMR (75 MHz, CDCl_3_, 25 °C): δ = 171.3, 137.3, 129.1, 128.4, 126.3, 43.2, 39.2, 14.1, 12.9 ppm.

## 4. Conclusions

In summary, a library of CDMT/*tert*-amine systems was investigated and their activity as dehydro-condensation agents for the production of amides compared to similar isolated quaternary ammonium salts synthesized by reaction of CDMT with a *tert*-amine (DMT-Ams(X), with X: Cl^−^ or ClO_4_^−^) was studies. In most cases, the CDMT/*tert*-amine systems prepared by simultaneous addition of a carboxylic acid, an amine, CDMT, and a *tert*-amine in the reaction solvent gave comparable or higher yields than the corresponding isolated DMT-Ams(X) species, both with Cl^−^ or ClO_4_^−^ as counter anion. In the presence of Cl^−^ as counter anion, only DMTMM could be synthesized and employed for dehydro-condensation reaction of **1a** and **2a**, while all corresponding CDMT/*tert*-amine system tested gave high yields in amide **3a**. Further studies are ongoing to gain deeper insight into the possible mechanistic differences between the two families of dehydro-condensation agents.

Moreover, CDMT/*tert*-amine may be used in alcohols or even water without any influence on the selectivity in the desired amide, adversely to other amidation protocols, which require less environmentally friendly and more expensive aprotic solvents.

The possibility to use CDMT/*tert*-amine systems allowed to achieve a library of easy and sustainable dehydro-condensation agents. Consequently, *tert*-amines could be chosen according to specific requirements such as availability, cost and risk assessment, and features, which may be crucial for industrial applications [71]. Additionally, amines such as NNDP, having two reactive *tert*-amine groups, allowed to reduce the consumption of the *tert*-amine up to 50%. Further advantage of both CDMT/*tert*-amine systems and DMTMM, compared to other dehydro-condensation agents commonly used today, is that triazine by-product formed during the reaction (DMTOH) is nontoxic and can be recovered and recycled to produce CDMT or DMTMM [26]. In summary, CDMT/*tert*-amine systems, formulated according to the specific protocol reported in this work, have been demonstrated to be very active, versatile, simple, and environmentally sustainable alternatives to many conventional dehydro-condensation agents employed today.

## Figures and Tables

**Table 1:** Dehydro-condensation reaction between **1a** and **2a** by 2-chloro-4,6-dimethoxy-1,3,5-triazine (CDMT)/*N*-methyl morpholine (NMM) system or isolated 4-(4,6-dimethoxy-1,3,5-triazin-2-yl)-4-methylmorpholinium (DMTMM) in different solvents.

Entry	Solvent	Coupling Agent ^[a]^	Yield (%) ^[b]^ (15/60 min)
1	THF	CDMT/NMM	74/78
2	THF	DMTMM	80/89
3	CH_3_OH	CDMT/NMM	93/95
4	CH_3_OH	DMTMM	98/99
5	EtOH	CDMT/NMM	92/95
6	EtOH	DMTMM	98/99
7	CH_3_CN	CDMT/NMM	78/82
8	CH_3_CN	DMTMM	58/66
9	Acetone	CDMT/NMM	82/83
10	Acetone	DMTMM	73/97
11	CH_2_Cl_2_	CDMT/NMM	86/93
12	CH_2_Cl_2_	DMTMM	85/92
13	Toluene	CDMT/NMM	69/90
14	Toluene	DMTMM	65/72
15	H_2_O	CDMT/NMM	45/52 ^[c]^
16	H_2_O	DMTMM	49/53 ^[c]^

Reaction conditions: benzoic acid: 1.3 mmol, phenylethylamine: 1.2 mmol, Solvent: 6 mL, T: 25 °C. ^[a]^ Coupling agent: 1.3 mmol of CDMT and 1.3 mmol of NMM were added; for isolated DMTMM, 1.3 mmol were used. ^[b]^ Yield of **3a** was measured by GLC using mesitylene as internal standard. ^[c]^ Yields were measured on weight of **3a** recovered after workup.

**Table 2:** Dehydro-condensation reaction between **1a** and **2a** with CDMT/*tert*-amine system or isolated 2-(4,6-dimethoxy-1,3,5-triazinyl)trialkyl ammonium salts (DMT-Ams).

Entry	Coupling Agent ^[a]^	Counter Anion ^[b]^	Yield (%) ^[c]^ (15/60 min)
1	CDMT/NMM	Cl^−^	93/95
2	DMTMM	Cl^−^	98/99
3	CDMT/NMM	ClO_4_^−^	82/83
4	DMTMM	ClO_4_^−^	38/47
5	CDMT/TMA	Cl^−^	93/96
6	DMTTMA ^[d]^	Cl^−^	n.d.
7	CDMT/TMA	ClO_4_^−^	87/91
8	DMTTMA	ClO_4_^−^	79/81
9	CDMT/NMP	Cl^−^	49/52
10	DMTMP ^[d]^	Cl^−^	n.d.
11	CDMT/NMP	ClO_4_^−^	56/60
12	DMTMP	ClO_4_^−^	86/90
13	CDMT/MPD	Cl^−^	65/77
14	DMTMPD ^[d]^	Cl^−^	n.d.
15	CDMT/MPD	ClO_4_^−^	68/80
16	DMTMPD	ClO_4_^−^	80/85
17	CDMT/NNDP	Cl^−^	93/96
18	CDMT/NNDP ^[e]^	Cl^−^	92/95
19	CDMT/NNDP ^[f]^	Cl^−^	79/90
20	DMTDP ^[d]^	Cl^−^	n.d.
21	CDMT/NNDP	ClO_4_^−^	77/88
22	DMTDP ^[d]^	ClO_4_^−^	n.d.

Reaction conditions: benzoic acid: 1.3 mmol, phenylethylamine: 1.2 mmol, solvent: CH_3_OH (6 mL), T: 25 °C. ^[a]^ Coupling agent: 1.3 mmol of CDMT and 1.3 mmol of tertiary amine were added, for isolated DMT-Ams, 1.3 mmol of were added. ^[b]^ For the CDMT/*tert*-amine/NaClO_4_ system, 1.3 mmol of CDMT, 1.3 mmol of NaClO_4_, and 1.3 mmol of tertiary amine were added, while of the isolated system, 1.3 mmol of DMT-Ams(ClO_4_) were added to the reaction mixture. ^[c]^ Yield in **3a** was measured by GLC using mesitylene as internal standard. ^[d]^ Not determined since the isolated DMT-Ams(Cl) was not isolated. ^[e]^ For this, 0.75 eq of *tert*-amine used. ^[f]^ For this, 0.5 eq of *tert*-amine used.

**Table 3:** Dehydro-condensation reaction of **2a** and a carboxylic acid (**1a–1e**) by CDMT/*tert*-amine system.

Entry	Acid	Coupling Agent ^[a]^	Amide	Yield (%) ^[b]^ (15/60 min)
1	**1a**	CDMT/NMM	**3a**	93/95
2		CDMT/NNDP		92/95
3		CDMT/TMA		93/96
4	**1b**	CDMT/NMM	**3b**	82/87
5		CDMT/NNDP		69/71
6		CDMT/TMA		45/51
7	**1c**	CDMT/NMM	**3c**	71/75
8		CDMT/NNDP		65/74
9		CDMT/TMA		12/15
10	**1d**	CDMT/NMM	**3d**	88/92
11		CDMT/NNDP		70/86
12		CDMT/TMA		18/21
13	**1e**	CDMT/NMM	**3e**	34/38

Reaction conditions: phenethylamine **2a**: 1.2 mmol, carboxylic acid (**1a**–**1e**): 1.3 mmol, solvent: CH_3_OH (6 mL), T: 25 °C. ^[a]^ Coupling agent: 1.3 mmol of CDMT and 1.3 mmol of tertiary amine. ^[b]^ Yields were measured by GLC using mesitylene as internal standard.

**Table 4:** Dehydro-condensation reaction of **1a** and an amine (**2b**–**2e**) by CDMT/*tert*-amine system.

Entry	Amine	Coupling Agent ^[a]^	Amide	Yield (%) ^[b]^ (15/60 min)
1	**2b**	CDMT/NMM		58/74
2		CDMT/NNDP	**3f**	49/65
3		CDMT/TMA		52/67
4	**2c**	CDMT/NMM		77/93
5		CDMT/NNDP	**3g**	59/64
6		CDMT/TMA		72/78
7	**2d**	CDMT/NMM		61/70
8		CDMT/NNDP	**3h**	55/58
9		CDMT/TMA		19/30
10	**2e**	CDMT/NMM		56/66
11		CDMT/NNDP	**3i**	50/55
12		CDMT/TMA		34/35

Reaction conditions: benzoic acid **1a**: 1.3 mmol, amine **2b**–**2e**: 1.2 mmol, solvent: CH_3_OH (6 mL), T: 25 °C. ^[a]^ Coupling agent: 1.3 mmol of CDMT and 1.3 mmol of tertiary amine. ^[b]^ Yields were measured by GLC using mesitylene as internal standard.

## Data Availability

Not applicable.

## Supplementary Materials

The Supplementary Materials are available online, Chapter I contains the NMR and FT-IR spectra of DMTTMA(ClO_4_) and Chapter II contains the ^1^H NMR of the dehydro-condensation reaction products and triazine derivatives monitored in time.

## Sample Availability

Samples of the compounds are available from the authors.
