# Supplementary material for: Sustainable Triazine-Based Dehydro-Condensation Agents for Amide Synthesis
Source: Molecules. 2021 Jan 2;26(1):191. doi: 10.3390/molecules26010191 (PMC7795458; doi:10.3390/molecules26010191)
Supplement: Supplementary file 1 [file molecules-26-00191-s001.pdf]

SUPPORTING INFORMATION

Sustainable Triazine Based Dehydro-condensation Agents for Amide Synthesis

by

R. Sole[1], V. Gatto[1,2], S. Conca[1], N. Bardella[1], A. Morandini[1], and V. Beghetto[1,2]*

[1] Università Ca' Foscari di Venezia, Dipartimento di Scienze Molecolari e Nanosistemi, Via Torino 155, 30172 Venezia (Italy);

[2] Crossing srl, Viale della Repubblica 193/b, 31100 Treviso (Italy); vanessa.gatto@crossing-srl.com, valentina.beghetto@crossing-srl.com

Table of contents

I. ^{1}H, ^{13}C NMR and FT-IR of 4-(4,6-dimethoxy-1,3,5-triazin-2-yl)-4-trimethylamine perchlorate (DMTTMA(ClO_4))

II. NMR of reaction products

I. ^{1}H, ^{13}C NMR and FT-IR of 4-(4,6-dimethoxy-1,3,5-triazin-2-yl)-4-trimethylamine perchlorate (DMTTMA(ClO_4))

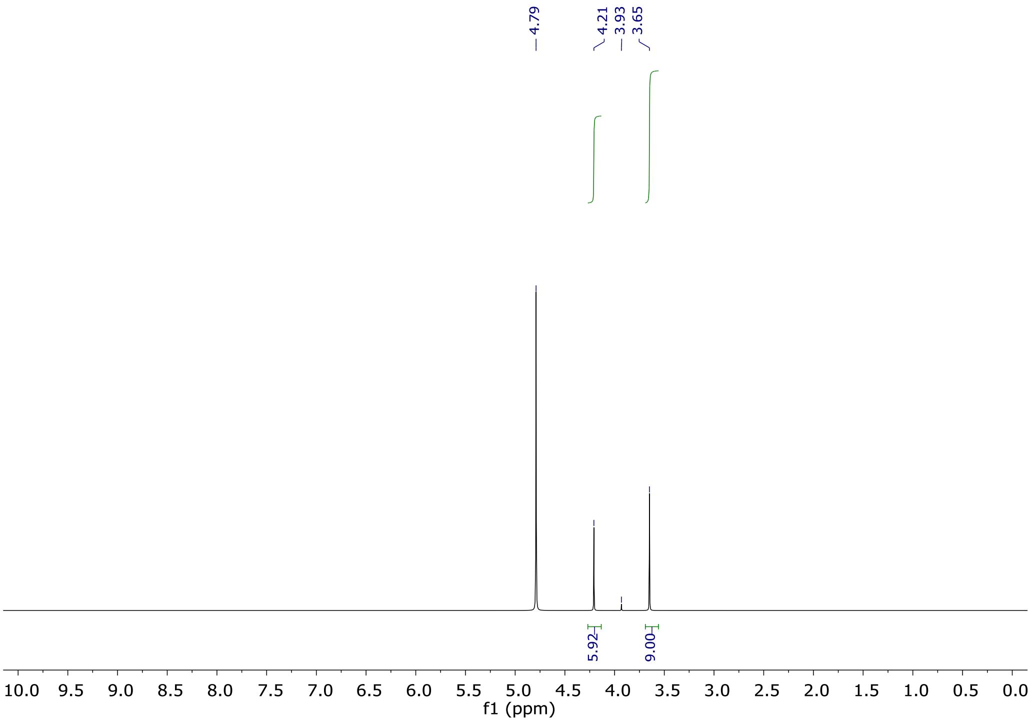

^{1}H NMR (300 MHz, D_2O, 25°C): δ=4.21 (s, 6H), 3.65 ppm (s, 9H)

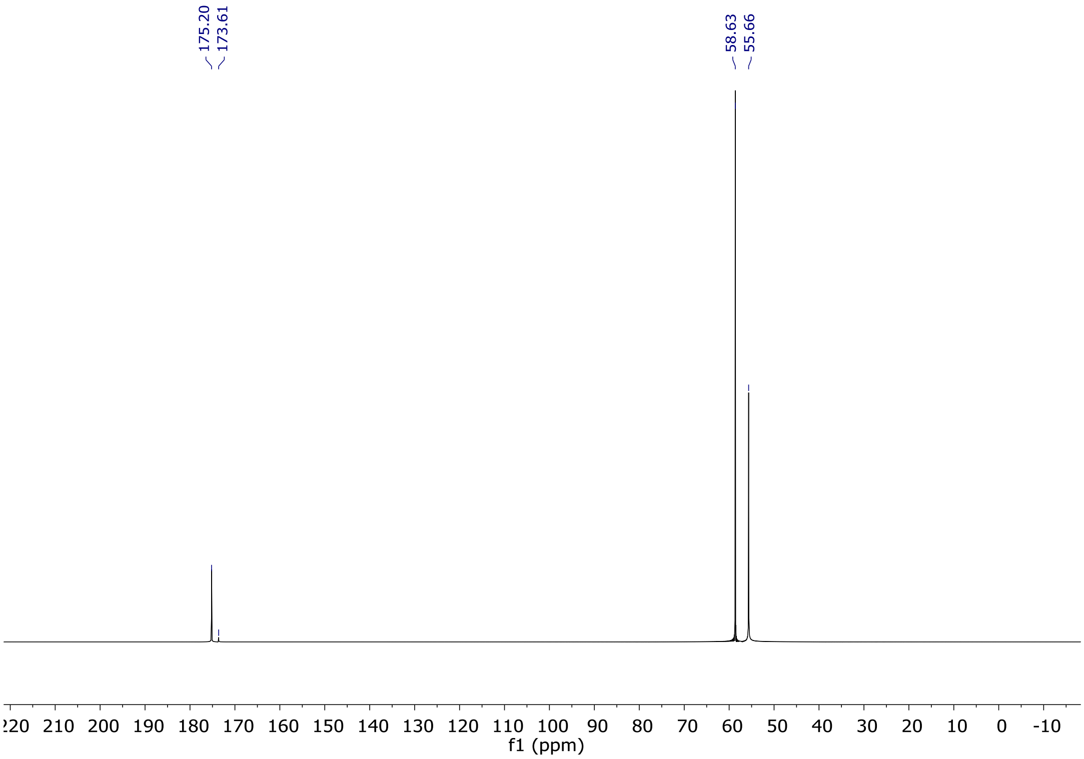

^{13}C NMR (75 MHz, D_2O, 25°C): δ=175.2, 173.6, 58.6, 55.7 ppm

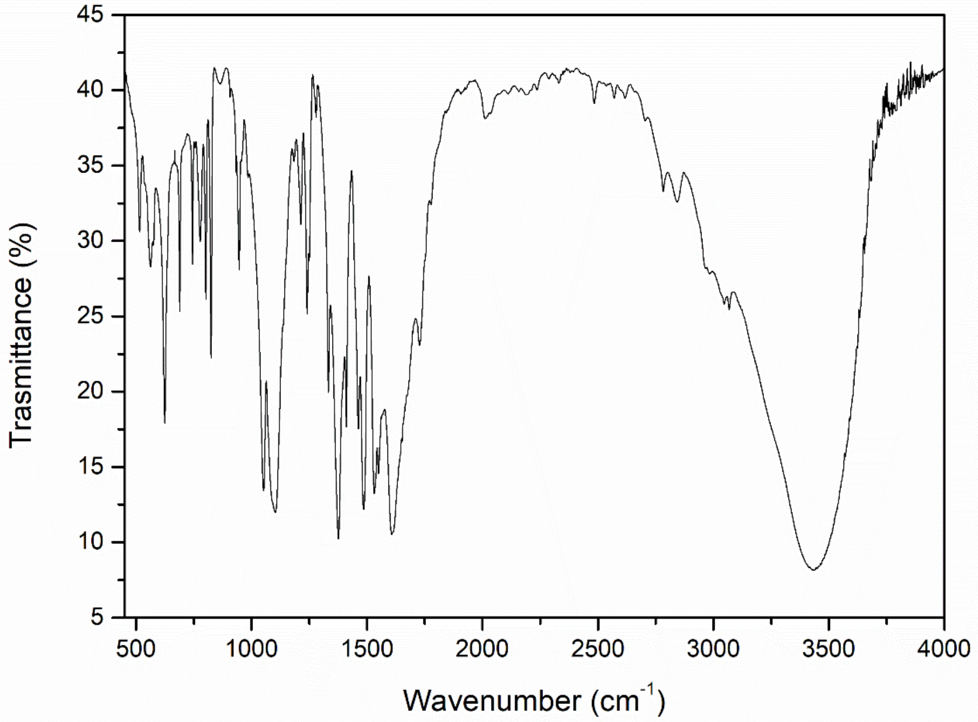

FT-IR (KBr): ṽ=1606, 1531, 1485, 1375, 1052, 311 825, 624 cm^{-1}

II. NMR study of reaction products

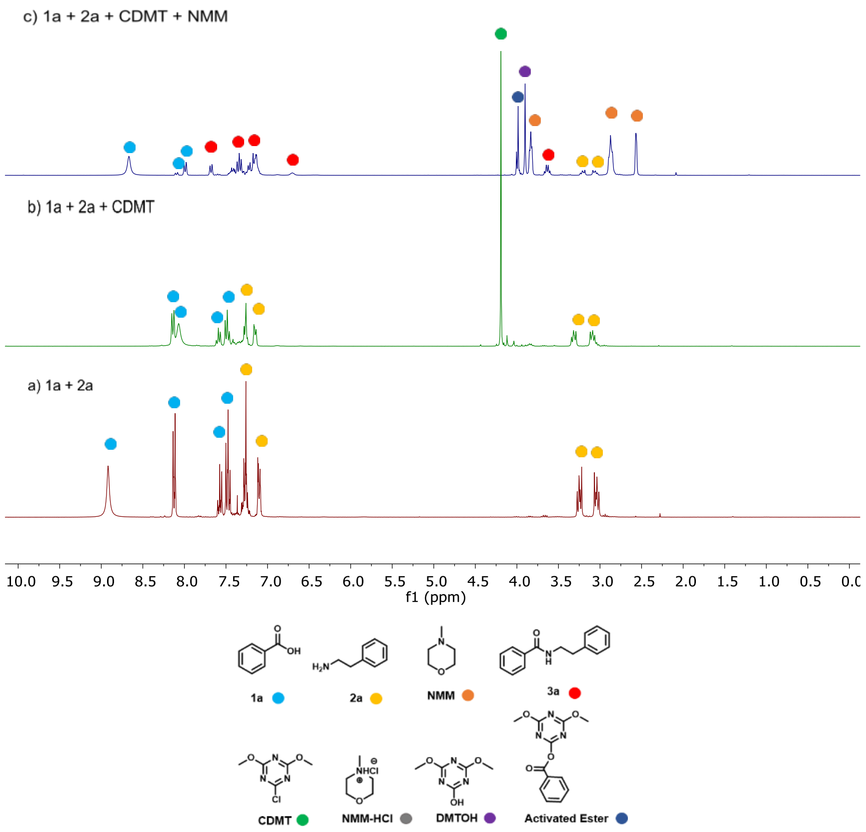

^{1}H NMR spectra ($CDCl_3$) of a) benzoic acid **1a** and phenylethylamine **2a** b) **1a** + **2a** + CDMT c) **1a** + **2a** + CDMT + NMM.

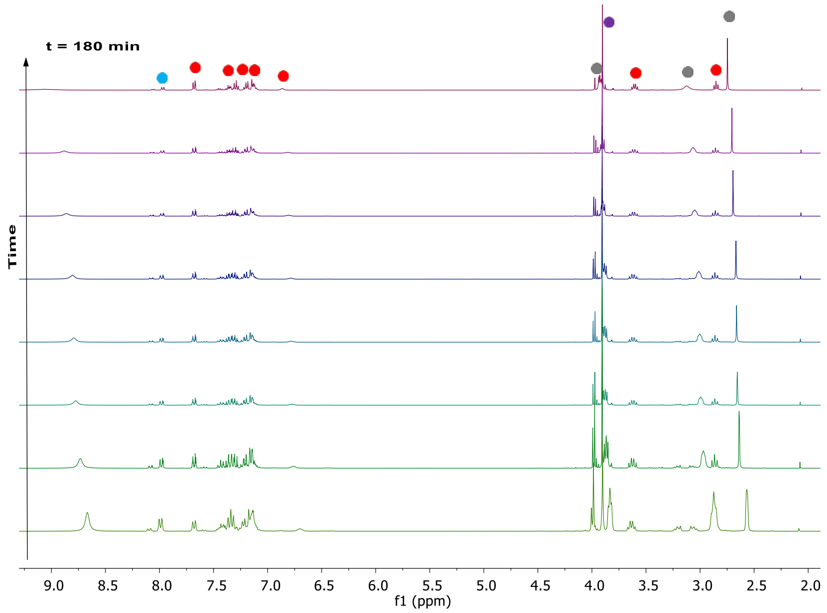

^{1}H NMR spectra ($CDCl_3$) in sequence over the time of **1a** + **2a** + CDMT + NMM, t = 0 at the bottom, t = 180 min at the top.
